# Systemic fluoroquinolone prescriptions for hospitalized children in Belgium, results of a multicenter retrospective drug utilization study

**DOI:** 10.1186/s12879-018-2994-z

**Published:** 2018-02-23

**Authors:** Kevin Meesters, Reiner Mauel, Evelyn Dhont, Johan Vande Walle, Pauline De Bruyne

**Affiliations:** 1 Department of Pediatrics, Vrije Universiteit Brussel (VUB), Universitair Ziekenhuis Brussel (UZ Brussel), Laarbeeklaan 101, 1090 Brussels, Belgium; 2 Department of Pediatrics, Ghent University Hospital, De Pintelaan 185, 9000 Ghent, Belgium; 3 Pediatric Intensive Care Unit, Ghent University Hospital, De Pintelaan 185, 9000 Ghent, Belgium

**Keywords:** Fluoroquinolones, Children, Drug utilization study, Off-label prescribing, Prescription errors, Antimicrobial stewardship

## Abstract

**Background:**

Fluoroquinolones (FQ) are increasingly prescribed for children, despite being labeled for only a limited number of labeled pediatric indications. In this multicenter retrospective drug utilization study, we analyzed indications for systemic FQ prescriptions in hospitalized children and the appropriateness of the prescribed dose.

**Methods:**

Using data obtained from electronic medical files, the study included all children who received a systemic FQ prescription in two Belgian university children’s hospitals between 2010 and 2013. Two authors reviewed prescribed daily doses. Univariate and multivariate logistic regression models were used to analyze risk factors for inadequately dosing.

Results262 FQ prescriptions for individual patients were included for analysis. 16.8% of these prescriptions were for labeled indications, and 35.1% were guided by bacteriological findings. Prescribed daily dose was considered to be inappropriate in 79 prescriptions (30.2%). Other FQ than ciprofloxacin accounted for 9 prescriptions (3.4%), of which 8 were correctly dosed. Underdosing represented 45 (56.9%) dosing errors. Infants and preschool children were at particular risk for dosing errors, with associated adjusted OR of 0.263 (0.097–0.701) and 0.254 (0.106–0.588) respectively.

**Conclusions:**

FQ were often prescribed off-label and not guided by bacteriological findings in our study population. Dosing errors were common, particularly in infants and preschool children. FQ prescriptions for children should be improved by specific pediatric antimicrobial stewardship teams. Furthermore, pharmacokinetic studies should optimise dosing recommendations for children.

## Background

The cornerstone of an adequate antimicrobial prescription is the selection of the right drug for the targeted disease, in a correct dose, and administered via an appropriate route of delivery. Prescribing fluoroquinolones (FQ) for children is controversial as labeled pediatric indications for FQ prescription are limited. Moreover, the safety of systemic FQ for growing children has been debated for a long time [1, 2]. Ciprofloxacin, the prototype FQ, is labeled in the United States (US) for treating complicated urinary tract infections (UTI), and treatment and prevention of inhalation anthrax in children [3]. In Europe, ciprofloxacin is labeled for the abovementioned indications, and additionally for treatment of *Pseudomonas aeruginosa* infections in cystic fibrosis (CF) patients [4]. Furthermore, both labeling guidelines state that beyond these indications, FQ should be reserve antibiotics for children in case of difficult to treat infections in absence of other available agents [3, 4]. Nevertheless, 520,000 children were prescribed a FQ in the USA in 2002 [5], the most recent year for which data could be found. Prescribing FQ for children has some advantages, even in the absence of indications outlined in labeling guidelines. First, FQ cover a broad spectrum of bacteriae [6]. Second, pharmacokinetic (PK) characteristics of systemic FQ are favorable. The bioavailability of common FQ agents is usually high and FQ typically penetrate in deep compartments [7–9]. These PK properties can be of particular interest in pediatric pharmacotherapy, as intravenous access is challenging in children, creating a higher need for oral alternatives. Further, microbial cultures are usually not easy to obtain in children, resulting in empiric treatments. However, the safety of FQ for growing children has been debated, as experiments in different juvenile animals showed irreversible cartilage tissue damage after exposure to systemic FQ [10–12]. These findings likely influence both the labeling guidelines and prescribers behavior, although there is no evidence of significant irreversible musculoskeletal side effects resulting from FQ use in children [13, 14]. Another concern in liberally prescribing FQ for children is the rapidly growing resistance rates of different germs for FQ [15, 16]. In this retrospective multicenter drug utilization study, we aimed to investigate indications for FQ prescription in a population of children hospitalized in two Belgian university children’s hospitals. Additionally, another goal was to assess the adequacy of prescribed doses, and risk factors for incorrectly dosed FQ prescriptions within this population.

## Methods

### Setting

The Department of Pediatrics at Ghent University Hospital is a tertiary referral hospital for the Western region of Belgium that admits around 4000 children each year. Universitair Ziekenhuis Brussel is the hospital of the Dutch-speaking university of Brussels (Vrije Universiteit Brussel), and serves as a tertiary referral hospital for the Brussels region. Its pediatric department admits about 2500 children annually. Both university children’s hospitals include all different subspecialities. During the study period, no hospital formulary of FQ dosing for either normal weight or obese children was available in the study centers. Children were dosed on total body weight. Furthermore. not all antibiotic prescriptions were reviewed by an antimicrobial stewardship (AMS) program. However, treating physicians usually used either the Dutch Paediatric Formulary, or labeling guidelines for dosing recommendations. Current pediatric dosing recommendations of FQ in the study centers are shown in Table 1.

**Table 1:** recommended doses of systemic FQ in the study centers

FQ	Adequate oral dose	Adequate intravenous dose
Ciprofloxacin	*non-*CF: 20–30 mg/kg/day in 2 doses, max. 500 mg/dose. CF: 40 mg/kg/day in 2 doses, max. 750 mg/dose	*non-*CF: 20–30 mg/kg/day in 2–3 doses, max. 400 mg/dose. CF: 30–40 mg/kg/day in 2–3 doses, max. 1200 mg/day.
Moxifloxacin	10 mg/kg/day in one dose, max. 400 mg/dose.	
Levofloxacin	*< 5 years*: 20 mg/kg/day in 2 doses, max. 1000 mg/day.	
	*> 5 years*: 10 mg/kg/day in 1 dose, max. 1000 mg/day.	
Norfloxacin	*Therapeutic indications*: 10 mg/kg/day in one dose, max. 400 mg/dose.	
	*Prophylactic indications*: 2–5 mg/kg/day in 2 doses, max. 400 mg/dose.	

### Participants

The pharmacy information system retrieved all FQ prescriptions for hospitalized children between 2010 and 2013 at both study centers. Information about the patients, indications and other details of their FQ prescription, and results of microbial cultures were obtained from the electronic medical files. For patients who were hospitalized repeatedly during the study period, only the first prescription per indication was included.

### Data classification

Participants older than 2 years of age were classified as being overweight or obese, if their body mass index (BMI) exceeded respectively the p85 or p95 of their sex and age specific reference value of Belgian children (Vrije Universiteit Brussel, 2004). If no length was available, participants were classified as overweight or obese if their weight exceeded the 85th centile for their age and sex specific curve. All prescriptions were classified as being either on-label or off-label according to the guidelines of the European Medicines Agency (EMA) [4]. More precisely, on-label indications are respiratory infections in CF patients, complicated UTI and pyelonephritis, and treatment and prophylaxis of inhalation anthrax. The first and second author independently assessed the prescribed daily doses per patient. Recommended doses of FQ in our centers are summarized in Table 1, and were obtained from available studies in either labeling leaflets or the Dutch Paediatric Formulary. Underdosing was defined, in absence of formal definitions, as a dose per kilogram of at least 5% below the minimum recommended dose per kilogram, while not exceeding the maximum dose. Similarly, overdosing was defined of a dose per kilogram that exceeds at least 5% of the maximum recommended dose per kilogram. Discrepancies between the two reviewers were resolved by the last author.

### Data analysis

Continuous data are presented as median with interquartile range, if not normally distributed, or as mean with 95% confidence interval if normally distributed. Categorical data are expressed as either frequencies, fractions, or percentages, unless stated otherwise. Chi-square tests were used to test differences in proportions of categorical variables between subgroups. Univariate logistic regression models were used for analyzing risk factors for inadequate prescriptions. A multivariate logistic regression model was used to assess interaction between different predictors. Appropriateness of prescribed dose, classified as described previously, was used as outcome variable in all regression analyses. Age was stratified according to different development stages: infants (< 1 year), toddlers (12 months–36 months), preschool children (3–6 years), school children (6–12 years), and young adolescents (12–18 years). Akaike Information Criterion was used for assessing quality of the multivariate logistic regression model. A p-level of less than 0,05 was considered statistically significant in all analyses. Data were analyzed using SPSS for Windows version 23 (SPSS Inc., Chicago, IL).

## Results

A total of 262 FQ prescriptions for unique patients were identified within the study period, of which 158 (60.3%) were at Ghent University Hospital. Table 2 displays basic characteristics of our study population. Length was unavailable for 21 participants older than 2 years of age, so that they were classified as being overweight or obese based on their age and sex specific weight curve.

**Table 2:** characteristics of the study population

Age	5.23 (1.75–12.44) *median, interquartile range*
	< 1 years: 26 (9.9%)
	1–3 years: 65 (24.8%)
	3–6 years: 43 (16.4%)
	6–12 years: 54 (20.6%)
	12–18 years: 74 (28.2%)
Weight	18,6 kg (11.3–38.7) *median, interquartile range*
BMI^a^	16.44 (15.00–18.76) *median, interquartile range*
Fraction overweight or obese^a^	1–3 years #: 0/20
	3–6 years: 7/43
	6–12 years: 5/54
	12–18 years:11/74
Study center	Ghent University Hospital: 158 (60.3%)
	Universitair Ziekenhuis Brussel: 104 (39.7%)
Sex	Male 144 (55%), female 118 (45%)
Comorbidity	Malignancies: 53 (20.2%) *of which hematologic: 39 (73.6%)*
	Cystic fibrosis: 14 (5.3%) Benign blood disorders and immune deficiencies: 14 (5.3%)
	Neurologic disorder: 38 (14.5%)
	Congenital Anomalies of the Kidneys and Urinary Tract: 18 (6.9%)
	Inflammatory bowel disease: 7 (2.7%)
	Heart defect: 3 (1.1%)
	Transplant organ: 3 (1.1%)
	Congenital hernia diaphragmatica: 1 (0.4%)
	None: 111 (42.4%)
Department	Academic pediatrics ward: 104 (39.7%)
	Pediatric oncology: 53 (20.2%)
	PICU: 105 (40.1%)

^a^Defined as BMI exceeding respectively p85 or p95 for age and sex specific curves for Belgian children.

#Starting for children of 2 years and older

Most children (57.6%) had significant chronic comorbidity such as any type of cancer (20.2%), a neurologic disease (14.5%), or congenital anomalies of the kidneys and urinary tract (CAKUT; 6.9%). Table 2 summarizes details about the FQ prescriptions. Ciprofloxacin was by far the most frequently prescribed FQ, representing 253 prescriptions (96.6%). Other prescribed FQ were moxifloxacin (1.1%), norfloxacin (0.4%), and levofloxacin (1.9%). Main indications for FQ prescription were treatment of central nervous system (CNS) infections (65 prescriptions, 24.8%), prophylaxis of febrile neutropenia (49 prescriptions, 18.7%), respiratory tract infections and pneumonias (48 prescriptions, 18.3%), and UTI (31 prescriptions, 11.8%) (Table 3). Overall, the number of on-label FQ prescriptions was 43 (16.4%). Prescription was guided by a microbial culture in 62 cases (35.1%), indicating empiric treatments in the majority of our study population. 79 prescriptions (30.2%), of which 78 were ciprofloxacin prescriptions, were considered to be inaccurately dosed. Underdosing was the most common type, as 57.1% of all inaccurately dosed prescriptions were underdosed. The percentage of underdosing was similar in our population of overweight and obese children compared to normal weight children (17.9% vs. 17.4%, *p* = 0.956).

**Table 3:** characteristics of the prescriptions

FQ prescribed	Ciprofloxacin: 253 (96.6%)
	Moxifloxacin: 3 (1.1%)
	Levofloxacin: 5 (1.9%)
	Norfloxacin: 1 (0.4%)
Main indication	On-label
	Respiratory infections in CF patients: 13 (5.0%)
	Complicated UTI and pyelonephritis: 31 (11.8%)
	Off-label
	Prophylaxis of febrile neutropenia: 49 (18.7%)
	Pneumonia: 34 (13.0%)
	Multidrug-resistant tuberculosis: 1 (0.4%)
	Sepsis: 6 (2.3%)
	Ocular trauma: 1 (0.4%)
	Intraabdominal abcess and peritonitis: 15 (5.7%)
	Enteritis: 13 (5.0%)
	Meningitis and/or encephalitis: 65 (24.8%)
	Epididymitis: 2 (0.8%)
	Skin and soft tissue infections: 27 (10.3%), *including burns and surgical site infections*
	Osteomyelitis: 2 (0.8%)
	Q-fever: 1 (0.4%)
Microbial cultures	None: 170 (64.9%)
	Pseudomonas aeruginosa: 51 (19.5%)
	Colonic bacteriae: 17 (6.5%)
	(*Escherichia coli*, Enterobacter cloaca, Citrobacter freundi)
	Stenotrophomonas maltophilia: 7 (2.7%)
	Salmonalla species (blood culture): 3 (1.1%)
	*Klebsiella pneumoniae*: 4 (1.5%)
	Mycoplasma pneumoniae: 2 (0.8%)
	Mycobacterium tuberculosis: 1 (0.4%)
	Acinetobacter species: 1 (0.4%)
	Proteus mirabilis: 1 (0.4%)
	Neiseria meningitidis, serogroup B: 1 (0.4%)
	Morganella morgani: 1 (0.4%)
Route of administration	Orally: 130 (49.6%)
	IV: 132 (50.4%)
Doses per day	1: 5 (1.9%)
	2: 240 (91.6%)
	3: 17 (6.5%)
Dose adequate:	Adequate: 183 (69.8%)
	Inadequate: 79 (30.2%)
	Underdosing: 45 (56.9%)
	Overdosing: 25 (31,6%)
	Unnecessarily thrice daily dosing PO: 9 (11,4%)

Table 4 displays the results of logistic regression analyses, aimed at identifying risk factors for dosing errors. In the univariate logistic regression analysis, children younger than 6 years of age were at particular risk of receiving an inadequately dosed prescription. Children who were prescribed an FQ other than ciprofloxacin, had a statistically non-significant odds ratio (OR) of 3.56 for receiving an adequately dosed prescription. Children treated both intravenously and for an off-label indication, were at risk for an inadequately dosed prescription. However, these associated OR were not statistically significant. Other potential risk factors were also not statistically significant in the univariate analyses. In the final multivariate logistic regression model, when controlled for the sort of FQ prescribed, OR for infants and preschool children remained statistically significant.

**Table 4:** risk factors for receiving an inadequately dosed FQ prescription

	Unadjusted Odds Ratio	Adjusted Odds Ratio
Age		
< 1 years	0.249 (0.092–0.658) *p* = 0.005	0.263 (0.097–0.701) *p* = 0.008
1–3 years	0.447 (0.198–0.976) *p* = 0.046	0.467 (0.206–1.027) *p* = 0.062
3–6 years	0.269 (0.113–0.622) *p* = 0.002	0.254 (0.106–0.588) *p* = 0.020
6–12 years	0.672 (0.281–1.605) *p* = 0.368	0.702 (0.292–1.684) *p* = 0.425
12–18 years	- (reference category)	- (reference category)
CF vs. non-CF	1.332 (0.398–3.992) *p* = 0.618	–
IV vs. orally	0.816 (0.480–1.384) *p* = 0.451	–
Study center	1.294 (0.753–2.253) *p* = 0.356	–
Off-label vs. on-label indication	0.780 (0.357–1.597) *p* = 0.512	–
Culture absent vs. present	1.292 (0.744–2.226) *p* = 0.358	–
Other FQ vs. ciprofloxacin	3.566 (0.638–66.71) *p* = 0.234	2.601 (0.436–49.86) *p* = 0.382
Department		
Academic pediatrics ward	1.238 (0.686–2.248) *p* = 0.479	
Oncology	1.213 (0.595–2.549) *p* = 0.601	
PICU	- (reference category)	

Table 4: unadjusted and adjusted odds ratios of different predictors of inadequately dosed prescriptions. Displayed are odds ratios with their 95% confidence intervals and corresponding *p*-values. For age categories, the stratum 12–18 years is the reference stratum as inadequately dosed prescriptions are least frequent in this stratum.

## Discussion

In this multicenter retrospective drug utilization study, we analyzed prescriptions of systemic FQ for hospitalized children in two Belgian university children’s hospitals, covering a three-year period. Ciprofloxacin was by far the most frequently prescribed FQ in our study population, representing 96.6% of all prescriptions. FQ prescriptions in our population were off-label in 83.6%, and not guided by a microbial culture in 64.9% of cases. Off-label medication use is very common in children, as is widely argued by professional societies [17]. However, at least some evidence is available for the major groups of off-label indications in our study population.

CNS infections represent the largest group of off-label indications for FQ prescription in our study population. This group consists of children admitted to the PICU with suspicion of meningoencephalitis. Beta lactams, particularly cephalosporins, are used in many centers for empiric treatment of meningoencephalitis. Cephalosporins penetrate into cerebrospinal fluid (CSF) of inflamed meninges, and usually cover *Streptococcus pneumoniae*, *Neiseria meningitidis*, and *Haemophilus influenzae*, which are common bacteria that cause meningitis. Additionally, *Mycoplasma pneumoniae* accounts for 10–30% of all pathogens causing encephalitis [18]. All antibiotics that target the cell wall, such as beta lactams and glycopeptides, are ineffective for *Mycoplasma pneumoniae* as this germ lacks a cell wall. Diagnosis of *Mycoplasma pneumonia* is challenging, given the complexity of culturing *Mycoplasma* strains [19], resulting in detection rates of *Mycoplasma* antigens in CSF as low as 0–14% [20]. Macrolides, tetracyclines, and FQ are usually effective against *Mycoplasma pneumoniae*. Yet, neither macrolides nor tetracyclines penetrate into CSF. Furthermore, macrolide resistance of *Mycoplasma pneumoniae* is growing worldwide, while FQ resistance has not yet been reported [21]. Therefore, FQ are preferred in our centers, as they usually reach therapeutic concentrations in cerebrospinal fluid [7–9].

Prophylaxis of febrile episodes in neutropenic patients, usually under treatment for childhood cancer, was the second most frequent off-label indication for FQ prescription in our study population. Progress in medical research has significantly improved long-term prognosis of most types of childhood cancer. However, infections remain a leading cause of morbidity and mortality in these patients. A meta-analysis of randomized placebo-controlled trials, in adult neutropenic patients under treatment for solid tumors showed that FQ prophylaxis reduced febrile episodes and overall mortality [22]. Furthermore, a Cochrane review pooled data of seven trials involving 850 adult neutropenic patients, and found that prophylaxis with FQ when compared to trimethoprim-sulfamethoxazole resulted in equal outcomes, but fewer side effects and less resistance to the drugs after treatment [23]. This has made FQ prophylaxis part of the ‘standard of care’ for adult neutropenic patients in many centers worldwide. Fewer studies are available on prophylactic antibiotics for febrile neutropenia in children. Previous studies of trimethoprim-sulfamethoxazole, erythromycin, and amoxicillin-clavulanic acid did not show significantly improved patient outcomes [24, 25]. To our knowledge only one RCT has investigated the value of FQ prophylaxis in neutropenic children [26]. In this Thai placebo controlled RCT, ciprofloxacin showed a statistically significant risk reduction of 23.0% (− 45.0% to − 0.9%) in febrile episodes in neutropenic children under treatment for acute leukemia. Still, no significant risk reduction for febrile episodes was observed, either in neutropenic children with lymphomas or in the consolidation phase of chemotherapy. No significant short-term adverse effects were observed, neither in the intervention group nor in the control group [26]. A retrospective study analyzed 153 chemotherapy courses in 45 neutropenic children under treatment for acute myeloid leukemia [27]. Sixty-four chemotherapy courses were under ciprofloxacin prophylaxis. This prophylaxis did not change the incidence of febrile or infectious episodes, or number of days of fever. Yet, ciprofloxacin prophylaxis was associated with a significant decrease in infections caused by Gram-negative germs and a concomitant significant increase in bacteremia’s caused by *Viridans* streptococci [27]. A concern about long-term consequences of prolonged FQ prophylaxis is increasing antimicrobial resistance, due to a selective pressure on intestinal flora. FQ resistance correlates with effective serum concentrations of FQ, at least in mycobacteriae [28]. For this reason, specific PK studies in children with cancer would be highly useful, as PK characteristics can be significantly influenced by age, maturational, and disease characteristics in children under treatment for cancer.

In our study population, 18 out of 34 non-CF patients for whom FQ were administered for pneumonias, had neurologic comorbidity such as cerebral palsies and muscular diseases. These patients, who often reside in specialized (para)medic institutions, suffer from complex pneumonias caused by health care associated pathogens, such as *Pseudomonas* and *Klebsiella* strains, which may justify FQ prescription. Ten of 34 patients with pneumonia had no significant medical history. Reasons for FQ administration to them were possibly empiric treatment of both typical and atypical germs. As mentioned before, macrolides, and tetracyclines are usually preferred when treating atypical germs, but may be unwanted because of rising antimicrobial resistance rates and adverse effects. Moxifloxacin was administered for one participant for treating multidrug-resistant tuberculosis (MDR-TB). In adults, FQ are widely used in treatment of MDR-TB [29]. FQ achieve antimicrobial activity by interfering with *mycobacterial gyrase* enzymes [28]. No prospective RCT has, to our knowledge, proven efficacy of FQ treatment for MDR-TB in children. Though, resistance patterns of mycobacteriae emerge rapidly, probably due to mutations in *gyr A* genes. The rate of mutations depends on effective FQ concentrations. There is a paucity on specific PK studies in MDR-TB infected children, especially those younger than 5 years of age, and HIV infected children. Yet, a South African study showed substantially lower serum concentrations of moxifloxacin after oral administration, when compared to adults [28], probably due to faster elimination in children.

Treatment of skin- and soft tissue infections account for 10.3% of our analyzed prescriptions. Usually, germs as *Staphylococcus aureus* and *Streptococcus pyogenes* are responsible for these infections, and topical antibiotics are preferred for less complicated infections. If systemic antibiotics are indicated, then flucloxacillin, amoxicillin-clavulanic acid, trimethoprim-sulfamethoxazole, and clindamycin are first line therapies for these infections [25]. Linezolid and vancomycin should be reserved for more severe or hospital acquired infections [25]. Yet, skin and soft tissue infections in our study population were often very complicated, as for example infected burns caused by *Pseudomonas* strains. In a skin microdialysis study in healthy male volunteers, systemic ciprofloxacin reached sufficient antimicrobial activity against *Pseudomonas aeruginosa* in the skin [30, 31]. No study has, to our knowledge, investigated skin penetration of FQ in children with complicated skin infections.

Bioavailability of most FQ is high after oral administration. Nevertheless, approximately half of our study population (50.4%) received their FQ intravenously. As the majority of our study population were hospitalized in advanced care departments, being either the PICU or the pediatric oncology unit, it is likely that clinical conditions such as vomiting, mucositis and oral ulcers, or significant respiratory distress, required intravenous treatment, because oral ingestion was not reliable. Yet, alternative antibiotics should be considered on a case by case basis if the intravenous route is used, for reasons of antimicrobial resistance and financial considerations.

Approximately a third (30.2%) of FQ prescriptions in our study population were considered inadequately dosed. Different information systems such as the Dutch Paediatric Formulary [32] and the Stanford Guide to Antimicrobial Therapy were available in the centers during the study period, but no pediatric hospital formulary for FQ was available. All except one inadequately dosed prescriptions were ciprofloxacin prescriptions. In both univariate and multivariate logistic regression models, prescription of a non-ciprofloxacin FQ was associated with an OR of about 3 in favor of receiving an adequately prescribed dose. One explanation for this might be that due to the small amount of levofloxacin, moxifloxacin, and norfloxacin prescriptions in our population, prescribers might have been more cautious in prescribing these non-ciprofloxacin FQ, resulting in a higher likelihood of prescribing a correct dose. However, the corresponding 95% confidence interval is very wide and therefore statistically insignificant, most likely due to the relatively low number of non-ciprofloxacin prescriptions. In our multivariate logistic regression model, age was statistically the most significant risk factor for receiving an inadequately dosed FQ prescription. Dosing errors were least frequent in adolescents, most likely due to similarities with widely available adult dosing recommendations. Infants and preschool children were at particular risk for dosing errors, with adjusted OR of 0.263 and 0.254 respectively. In both study centers, ciprofloxacin is available both in a liquid formulation, and in tablets of 250 mg and 500 mg. The liquid formulation was prescribed for all infants, whereas a combination of split tablets and liquid formulation was prescribed for preschool children. A rounding-off effect possibly explains a proportion of inadequately prescribed doses for children in these age categories.

Underdosing was the most common type of dosing error, and is undesirable as subtherapeutic serum concentrations of FQ result in rapidly emerging resistance rates, at least in mycobacteria [28] and *Pseudomonas aeruginosa* [33]. There is no universal definition of underdosing. Yet, in absence of high level pharmacokinetic studies in children, the prescribed dose per kilogram of FQ should at least be within the recommended range if the maximum recommended dose has not been reached. Different studies show that even advised doses per kilogram of different antibiotics in children are excreted faster than in adults, as has been shown for moxifloxacin [28] and for amoxicillin-clavulanic acid [34]. Therefore, we chose a sharp criterion for underdosing, i.e. a dose per kilogram of less than 95% of the minimum advised dose.

Overweight and obesity are increasingly prevalent among children, which creates specific dilemmas in drug dosing [35]. There were no specific recommendations available for FQ dosing for overweight and obese children in our centers, during the study period. Furthermore, there is no consensus on which body size descriptor should be used for dosing FQ in obese children. Some authors advocate dosing on total body weight, while others recommend dosing on metric that adjusts for body composition, such as adjusted body weight [36]. In our population, underdosing was similar between overweight and obese children, and normal weight children. Accordingly, overweight and obesity are unlikely to have influenced the proportion of underdosing in our study population.

Compared to a retrospective [37] and a prospective [38] study in Parisian children’s hospitals, our study showed a similar amount of off-label FQ prescriptions. Furthermore, frequent off-label indications for FQ prescription in these studies were prophylaxis of febrile neutropenia, intra-abdominal infections and pneumonia, all similar to our study population. The amount of correctly dosed prescriptions in our study was 69.8%, which is substantially lower than in these Parisian studies: 84.7% and 88% respectively [37, 38]. Not all antibiotic prescriptions were routinely reviewed by a formal AMS program. This does by no means justify dosing errors, as dosing is a joint responsibility of the prescriber and the hospital pharmacy. However, specific for antibiotics, drug dosing is important in achieving therapeutic success, and dosing can differ both on patient and disease characteristics, particularly in children. Feedback on the correct drug for a given indication, the dose, route of delivery, and treatment duration are key tasks of pediatric AMS programs [39]. Furthermore, a recent study in Germany showed an increase in dosage accuracy (78.8 vs. 97.6%) of different antibiotics after implementation of a pediatric AMS program [40]. Besides dose optimization, pediatric AMS programs in our hospitals could have reduced the number of FQ administered via the IV route, and have used other agents as much as possible.

This is, to our knowledge, the largest multicenter drug utilization study that analyzes FQ prescriptions for hospitalized children. A limitation of this study is its retrospective design, which makes it impossible to reliably study adverse effects of FQ administration. However, adverse effects of FQ in children have been extensively studied previously [13], and was for this reason not a part of our study protocol. Another possible limitation of retrospective study designs is information bias. Yet, the high quality of electronic medical files at both study sites guaranteed complete data extraction, leaving minimal missing values for our parameters. Further, as our study was conducted within two university children’s hospitals with important referral functions for complex pediatric pathologies and for critically ill children, our results are probably selection biased to tertiary pathologies.

## Conclusions

In our study, 83.2% of systemic FQ prescriptions were off-label prescribed for hospitalized children. Dosing errors were present in 30.2% of all prescriptions, with infants and preschool children at particular risk for receiving an inadequately dosed prescription. Our study results suggest that FQ prescriptions for hospitalized children should be optimized. AMS programs should control for inappropriate FQ prescriptions, and adjust inappropriate doses. These programs should focus on special populations at risk. Furthermore, pharmacokinetic studies are necessary for identifying optimal doses for frequent off-label indications.

## Abbreviations

AMS: Antimicrobial stewardship
BMI: Body mass index
CAKUT: Congenital anomalies of the kidneys and urinary tract
CF: Cystic fibrosis
CNS: Cerebral nervous system
CSF: Cerebrospinal fluid
EMA: European Medicines Agency
FDA: Food and Drug Administration
FQ: Fluoroquinolones
MDR-TB: Multidrug-resistant tuberculosis
OR: Odds ratio
PICU: Pediatric intensive care unit
PK: Pharmacokinetic
US: United States
UTI: Urinary tract infection
